# *catena*-Poly[μ_2_-iodido-diiodidobis(μ_3_-pyridine-2-thione-κ^3^ *S*:*S*:*S*)(μ_2_-pyridine-2-thione-κ^2^ *S*:*S*)tricopper(I)]

**DOI:** 10.1107/S1600536812052051

**Published:** 2013-01-09

**Authors:** Fang Ke, Wen Wu

**Affiliations:** a College of Pharmacy, Fujian Medical University, Fuzhou 350004, People’s Republic of China; b Xiamen Maternity and Child Health Care Hospital, Xiamen 361001, People’s Republic of China

## Abstract

In the title compound, [Cu_3_I_3_(C_5_H_5_NS)_3_]_*n*_, a polymeric structure is formed along [100] through bridging iodide and pyridine-2-thione ligands. The metal atoms are engaged in [Cu_3_S_3_] and [Cu_2_S_2_] rings sharing Cu—S edges, with the [Cu_2_S_2_] rings located about inversion centers. Cu^I^ atoms bridged by iodide ions exhibit the shortest Cu⋯Cu separation in the polymer [2.8590 (14) Å]. The three independent Cu^I^ atoms all display distorted tetra­hedral coordination geometries.

## Related literature

For applications of Cu^I^ complexes and coordination compounds based on 1*H*-pyridine-2-thione, see: Kitagawa *et al.* (1990 ▶); Raper (1996 ▶, 1997 ▶); García-Vázquez *et al.* (1999 ▶); Akrivos (2001 ▶); Lobana & Castineiras (2002 ▶). For the structure of a polymer isoformular to the title compound, see: Lobana *et al.* (2003 ▶).
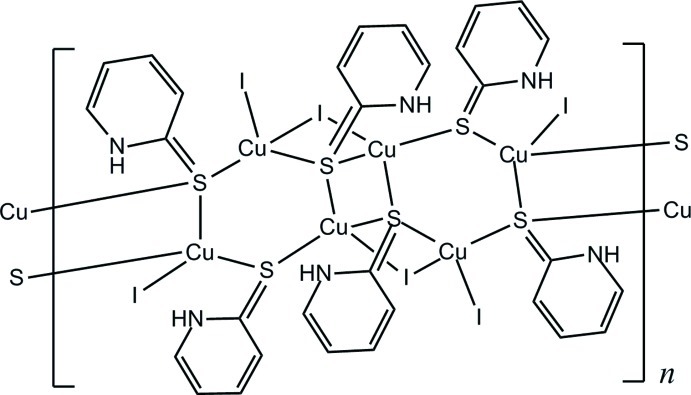


## Experimental

### 

#### Crystal data

[Cu_3_I_3_(C_5_H_5_NS)_3_]
*M* *_r_* = 904.80
Triclinic, 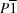

*a* = 10.035 (1) Å
*b* = 10.9220 (11) Å
*c* = 12.2500 (15) Å
α = 105.956 (2)°
β = 100.088 (3)°
γ = 109.377 (3)°
*V* = 1164.1 (2) Å^3^
*Z* = 2
Mo *K*α radiation
μ = 6.97 mm^−1^
*T* = 298 K
0.40 × 0.23 × 0.15 mm

#### Data collection

Bruker SMART CCD diffractometer
Absorption correction: multi-scan (*SADABS*; Sheldrick, 1996 ▶) *T*_min_ = 0.167, *T*_max_ = 0.421
6013 measured reflections
4009 independent reflections
3215 reflections with *I* > 2σ(*I*)
*R*_int_ = 0.028

#### Refinement

*R*[*F* ^2^ > 2σ(*F* ^2^)] = 0.040
*wR*(*F* ^2^) = 0.115
*S* = 1.06
4009 reflections
244 parameters
H-atom parameters constrained
Δρ_max_ = 1.33 e Å^−3^
Δρ_min_ = −1.45 e Å^−3^

### 

Data collection: *SMART* (Siemens, 1996 ▶); cell refinement: *SAINT* (Siemens, 1996 ▶); data reduction: *SAINT*; program(s) used to solve structure: *SHELXS97* (Sheldrick, 2008 ▶); program(s) used to refine structure: *SHELXL97* (Sheldrick, 2008 ▶); molecular graphics: *SHELXTL* (Sheldrick, 2008 ▶); software used to prepare material for publication: *SHELXTL*.

## Supplementary Material

Crystal structure: contains datablock(s) I, global. DOI: 10.1107/S1600536812052051/bh2469sup1.cif

Structure factors: contains datablock(s) I. DOI: 10.1107/S1600536812052051/bh2469Isup2.hkl

Additional supplementary materials:  crystallographic information; 3D view; checkCIF report

## Figures and Tables

**Table 1:** Selected bond lengths (Å)

Cu1—S3	2.315 (2)
Cu1—S1	2.356 (2)
Cu1—S1^i^	2.480 (2)
Cu1—I1	2.5917 (11)
Cu1—Cu2	2.8590 (14)
Cu1—Cu1^i^	2.928 (2)
Cu2—S2	2.315 (2)
Cu2—S1^i^	2.367 (2)
Cu2—I2	2.5632 (11)
Cu2—I1	2.6896 (12)
Cu3—S3	2.273 (2)
Cu3—S2^i^	2.290 (2)
Cu3—I3	2.5726 (11)
Cu3—S2^ii^	2.639 (3)

Symmetry codes: (i) 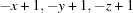; (ii) 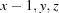.

**Fig. 1.**
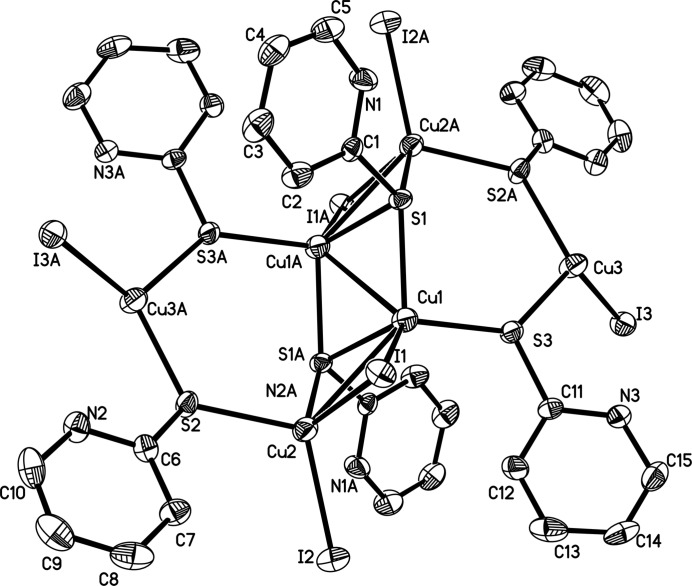
The molecular structure of the title compound, showing 30% probability displacement ellipsoids. H atoms have been omitted for clarity.

**Fig. 2.**
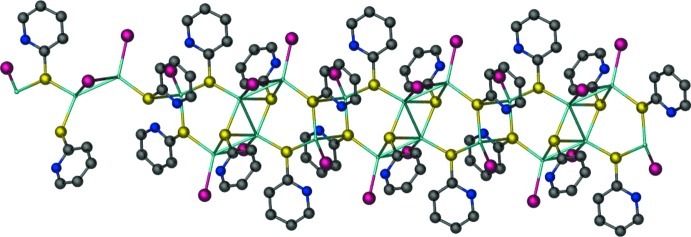
View of the one-dimensional extended chain structure in the title compound.

## supplementary crystallographic information

### Comment

The coordination chemistry of Cu(I) is of considerable interest because these complexes have luminescence properties, antimicrobial activity, and potential applications in catalysis, photography, and electrochemical processes (Kitagawa *et al.*, 1990; Raper, 1996, 1997). On the other hand, pyridine-2-thiolate can bind to a metal or a group of metals *via* a variety of bonding modes, and this versatility is attributed to the size of the S atom and its proximity to the pyridyl N atom (García-Vázquez *et al.*, 1999; Akrivos, 2001; Lobana *et al.*, 2002). The large size of the S atom makes it easier to adopt different coordination angles in complexes, which is necessary in order to match different geometries.

We report here the crystal structure of the title compound, which displays a polymeric chain structure (Fig. 1 and 2). The polymer is isoformular, although not isostructural to that previously described by Lobana *et al.* (2003). Two sulfur atoms S1 and S2 act as µ_3_-S donor atoms, while S3 acts as a µ_2_-S donor atom. There are three independent Cu atoms in the asymmetric unit; Cu1 and Cu3 are coordinated to S1, S3, I1, S1^i^ and S3, I3, S2^i^, S2^ii^, respectively. Atom Cu2 is coordinated to I1, I2, S2 and S1^i^ (symmetry codes: i: 1-*x*, 1-*y*, 1-*z*; ii: *x*-1, *y*, *z*). From bond lengths and angles around Cu centers, these metals are placed in a distorted tetrahedral coordination geometry. Moreover, metal–metal interactions are observed, with Cu1···Cu1^i^ and Cu1···Cu2 separations of 2.928 and 2.859 Å, shorter than metal–metal contacts in the isoformular polymer (Lobana *et al.*, 2003).

### Experimental

An oven-dried Schlenk tube was charged with CuI (0.4 mmol), and pyridine-2-thione (0.4 mmol). The tube was evacuated and backfilled with N_2_. The reaction mixture was stirred at 333 K for 4 h and then allowed to cool to room temperature. The insoluble residues were removed by filtration, and the filtrate was evaporated slowly at room temperature for about one month, to yield yellow crystals. Crystals suitable for single-crystal X-ray diffraction were selected directly from the sample as prepared.

### Refinement

All H atoms were placed in calculated positions and treated as riding on their parent atoms, with bond lengths fixed to 0.93 Å for C—H bonds and 0.86 Å for N—H bonds. Isotropic displacement parameters for H atoms were calculated as *U*_iso_(H) = 1.2*U*_eq_(carrier atom).

### Crystal data

**Table:**

[Cu_3_I_3_(C_5_H_5_NS)_3_]	*Z* = 2
*M**_r_* = 904.80	*F*(000) = 840
Triclinic, *P*1	*D*_x_ = 2.581 Mg m^−^^3^
Hall symbol: -P 1	Mo *K*α radiation, λ = 0.71073 Å
*a* = 10.035 (1) Å	Cell parameters from 3445 reflections
*b* = 10.9220 (11) Å	θ = 2.2–26.0°
*c* = 12.2500 (15) Å	µ = 6.97 mm^−^^1^
α = 105.956 (2)°	*T* = 298 K
β = 100.088 (3)°	Block, yellow
γ = 109.377 (3)°	0.40 × 0.23 × 0.15 mm
*V* = 1164.1 (2) Å^3^	

### Data collection

**Table:**

Bruker SMART CCD diffractometer	4009 independent reflections
Radiation source: fine-focus sealed tube	3215 reflections with *I* > 2σ(*I*)
Graphite monochromator	*R*_int_ = 0.028
φ and ω scans	θ_max_ = 25.0°, θ_min_ = 1.8°
Absorption correction: multi-scan (*SADABS*; Sheldrick, 1996)	*h* = −11→11
*T*_min_ = 0.167, *T*_max_ = 0.421	*k* = −12→12
6013 measured reflections	*l* = −14→10

### Refinement

**Table:**

Refinement on *F*^2^	Primary atom site location: structure-invariant direct methods
Least-squares matrix: full	Secondary atom site location: difference Fourier map
*R*[*F*^2^ > 2σ(*F*^2^)] = 0.040	Hydrogen site location: inferred from neighbouring sites
*wR*(*F*^2^) = 0.115	H-atom parameters constrained
*S* = 1.06	*w* = 1/[σ^2^(*F*_o_^2^) + (0.0556*P*)^2^ + 2.5913*P*] where *P* = (*F*_o_^2^ + 2*F*_c_^2^)/3
4009 reflections	(Δ/σ)_max_ < 0.001
244 parameters	Δρ_max_ = 1.33 e Å^−^^3^
0 restraints	Δρ_min_ = −1.45 e Å^−^^3^
0 constraints	

### Fractional atomic coordinates and isotropic or equivalent isotropic displacement parameters (Å^2^)

**Table:**

	*x*	*y*	*z*	*U*_iso_*/*U*_eq_	
Cu1	0.48634 (11)	0.50567 (10)	0.61815 (8)	0.0417 (3)	
Cu2	0.79614 (11)	0.58824 (10)	0.71789 (8)	0.0432 (3)	
Cu3	0.14805 (12)	0.65287 (11)	0.57965 (8)	0.0495 (3)	
I1	0.58333 (6)	0.39571 (5)	0.75952 (5)	0.04314 (17)	
I2	0.95579 (7)	0.79131 (6)	0.91260 (5)	0.0568 (2)	
I3	0.16049 (6)	0.90228 (5)	0.63917 (5)	0.04906 (18)	
N1	0.1790 (7)	0.0857 (6)	0.3003 (6)	0.0479 (17)	
H1	0.1089	0.1095	0.2766	0.057*	
N2	1.0746 (7)	0.3244 (7)	0.6514 (6)	0.0422 (15)	
H2	1.0476	0.2886	0.5751	0.051*	
N3	0.3203 (7)	0.7738 (6)	0.8801 (5)	0.0410 (15)	
H3	0.2341	0.7575	0.8377	0.049*	
S1	0.3069 (2)	0.35003 (18)	0.43695 (15)	0.0328 (4)	
S2	0.9240 (2)	0.4776 (2)	0.61847 (16)	0.0403 (5)	
S3	0.3492 (2)	0.6317 (2)	0.67672 (16)	0.0422 (5)	
C1	0.3003 (8)	0.1836 (7)	0.3837 (6)	0.0325 (16)	
C2	0.4146 (10)	0.1418 (8)	0.4198 (7)	0.047 (2)	
H2A	0.5022	0.2054	0.4778	0.057*	
C3	0.3937 (11)	0.0047 (9)	0.3675 (8)	0.055 (2)	
H3A	0.4690	−0.0231	0.3900	0.066*	
C4	0.2641 (11)	−0.0914 (8)	0.2828 (8)	0.053 (2)	
H4	0.2506	−0.1836	0.2491	0.063*	
C5	0.1579 (11)	−0.0491 (8)	0.2500 (9)	0.058 (2)	
H5	0.0695	−0.1122	0.1926	0.069*	
C6	1.0204 (8)	0.4156 (8)	0.7040 (6)	0.0360 (16)	
C7	1.0588 (9)	0.4641 (8)	0.8276 (7)	0.0438 (19)	
H7	1.0206	0.5237	0.8674	0.053*	
C8	1.1536 (11)	0.4235 (10)	0.8911 (8)	0.061 (3)	
H8	1.1787	0.4547	0.9736	0.073*	
C9	1.2119 (11)	0.3342 (11)	0.8293 (9)	0.063 (3)	
H9	1.2795	0.3095	0.8705	0.075*	
C10	1.1695 (10)	0.2871 (9)	0.7138 (9)	0.055 (2)	
H10	1.2054	0.2260	0.6729	0.066*	
C11	0.4100 (8)	0.7287 (7)	0.8249 (6)	0.0334 (16)	
C12	0.5518 (9)	0.7651 (8)	0.8966 (7)	0.045 (2)	
H12	0.6184	0.7385	0.8625	0.053*	
C13	0.5946 (11)	0.8381 (9)	1.0143 (8)	0.061 (3)	
H13	0.6904	0.8635	1.0603	0.074*	
C14	0.4937 (11)	0.8752 (9)	1.0667 (7)	0.056 (2)	
H14	0.5207	0.9219	1.1482	0.067*	
C15	0.3594 (11)	0.8436 (9)	0.9995 (7)	0.053 (2)	
H15	0.2922	0.8689	1.0337	0.064*	

### Atomic displacement parameters (Å^2^)

**Table:**

	*U*^11^	*U*^22^	*U*^33^	*U*^12^	*U*^13^	*U*^23^
Cu1	0.0459 (6)	0.0397 (5)	0.0395 (5)	0.0245 (5)	0.0052 (4)	0.0097 (4)
Cu2	0.0466 (6)	0.0423 (6)	0.0382 (5)	0.0235 (5)	0.0080 (4)	0.0064 (4)
Cu3	0.0618 (7)	0.0513 (6)	0.0355 (5)	0.0371 (5)	0.0002 (4)	0.0065 (4)
I1	0.0422 (3)	0.0431 (3)	0.0480 (3)	0.0177 (2)	0.0081 (2)	0.0245 (2)
I2	0.0619 (4)	0.0487 (4)	0.0412 (3)	0.0151 (3)	0.0040 (3)	0.0043 (3)
I3	0.0481 (3)	0.0371 (3)	0.0573 (4)	0.0208 (3)	0.0090 (3)	0.0095 (2)
N1	0.034 (4)	0.029 (3)	0.063 (4)	0.009 (3)	0.004 (3)	0.004 (3)
N2	0.051 (4)	0.043 (4)	0.047 (4)	0.030 (3)	0.016 (3)	0.024 (3)
N3	0.039 (4)	0.037 (3)	0.039 (3)	0.015 (3)	0.009 (3)	0.005 (3)
S1	0.0351 (9)	0.0281 (9)	0.0313 (9)	0.0152 (8)	0.0038 (7)	0.0058 (7)
S2	0.0494 (11)	0.0546 (12)	0.0292 (9)	0.0364 (10)	0.0111 (8)	0.0142 (8)
S3	0.0396 (11)	0.0502 (12)	0.0335 (10)	0.0285 (9)	0.0024 (8)	0.0026 (8)
C1	0.040 (4)	0.032 (4)	0.032 (4)	0.021 (3)	0.013 (3)	0.011 (3)
C2	0.058 (5)	0.038 (4)	0.042 (4)	0.023 (4)	0.005 (4)	0.012 (4)
C3	0.077 (6)	0.048 (5)	0.055 (5)	0.043 (5)	0.018 (5)	0.020 (4)
C4	0.075 (6)	0.029 (4)	0.058 (5)	0.026 (4)	0.023 (5)	0.013 (4)
C5	0.060 (6)	0.026 (4)	0.068 (6)	0.009 (4)	0.012 (5)	0.002 (4)
C6	0.037 (4)	0.040 (4)	0.040 (4)	0.019 (3)	0.014 (3)	0.021 (3)
C7	0.054 (5)	0.046 (5)	0.032 (4)	0.021 (4)	0.009 (3)	0.017 (3)
C8	0.066 (6)	0.066 (6)	0.037 (5)	0.010 (5)	−0.002 (4)	0.030 (4)
C9	0.056 (6)	0.072 (7)	0.076 (7)	0.032 (5)	0.006 (5)	0.049 (6)
C10	0.060 (6)	0.057 (6)	0.084 (7)	0.044 (5)	0.032 (5)	0.046 (5)
C11	0.041 (4)	0.023 (3)	0.032 (4)	0.012 (3)	0.007 (3)	0.007 (3)
C12	0.039 (4)	0.044 (5)	0.048 (5)	0.027 (4)	0.000 (3)	0.009 (4)
C13	0.063 (6)	0.049 (5)	0.060 (6)	0.029 (5)	−0.017 (5)	0.013 (4)
C14	0.079 (7)	0.045 (5)	0.026 (4)	0.012 (5)	0.005 (4)	0.007 (4)
C15	0.067 (6)	0.044 (5)	0.045 (5)	0.015 (4)	0.029 (5)	0.012 (4)

### Geometric parameters (Å, º)

**Table:**

Cu1—S3	2.315 (2)	S2—Cu3^i^	2.290 (2)
Cu1—S1	2.356 (2)	S2—Cu3^iii^	2.639 (3)
Cu1—S1^i^	2.480 (2)	S3—C11	1.708 (7)
Cu1—I1	2.5917 (11)	C1—C2	1.414 (10)
Cu1—Cu2	2.8590 (14)	C2—C3	1.384 (11)
Cu1—Cu1^i^	2.928 (2)	C2—H2A	0.9300
Cu2—S2	2.315 (2)	C3—C4	1.376 (13)
Cu2—S1^i^	2.367 (2)	C3—H3A	0.9300
Cu2—I2	2.5632 (11)	C4—C5	1.337 (13)
Cu2—I1	2.6896 (12)	C4—H4	0.9300
Cu3—S3	2.273 (2)	C5—H5	0.9300
Cu3—S2^i^	2.290 (2)	C6—C7	1.393 (10)
Cu3—I3	2.5726 (11)	C7—C8	1.385 (12)
Cu3—S2^ii^	2.639 (3)	C7—H7	0.9300
N1—C1	1.330 (9)	C8—C9	1.412 (14)
N1—C5	1.357 (10)	C8—H8	0.9300
N1—H1	0.8600	C9—C10	1.300 (13)
N2—C10	1.353 (10)	C9—H9	0.9300
N2—C6	1.354 (9)	C10—H10	0.9300
N2—H2	0.8600	C11—C12	1.396 (10)
N3—C11	1.355 (9)	C12—C13	1.351 (12)
N3—C15	1.365 (10)	C12—H12	0.9300
N3—H3	0.8600	C13—C14	1.399 (13)
S1—C1	1.728 (7)	C13—H13	0.9300
S1—Cu2^i^	2.367 (2)	C14—C15	1.326 (13)
S1—Cu1^i^	2.480 (2)	C14—H14	0.9300
S2—C6	1.721 (7)	C15—H15	0.9300
			
S3—Cu1—S1	95.70 (7)	Cu3^i^—S2—Cu3^iii^	87.07 (8)
S3—Cu1—S1^i^	109.08 (8)	Cu2—S2—Cu3^iii^	112.17 (9)
S1—Cu1—S1^i^	105.53 (6)	C11—S3—Cu3	112.4 (3)
S3—Cu1—I1	118.52 (7)	C11—S3—Cu1	113.8 (3)
S1—Cu1—I1	116.47 (6)	Cu3—S3—Cu1	133.81 (9)
S1^i^—Cu1—I1	110.13 (5)	N1—C1—C2	116.4 (6)
S3—Cu1—Cu2	126.14 (6)	N1—C1—S1	118.1 (5)
S1—Cu1—Cu2	135.86 (6)	C2—C1—S1	125.6 (6)
S1^i^—Cu1—Cu2	52.03 (5)	C3—C2—C1	118.9 (8)
I1—Cu1—Cu2	58.89 (3)	C3—C2—H2A	120.6
S3—Cu1—Cu1^i^	110.90 (7)	C1—C2—H2A	120.6
S1—Cu1—Cu1^i^	54.69 (6)	C4—C3—C2	121.5 (8)
S1^i^—Cu1—Cu1^i^	50.84 (5)	C4—C3—H3A	119.3
I1—Cu1—Cu1^i^	130.56 (5)	C2—C3—H3A	119.3
Cu2—Cu1—Cu1^i^	93.24 (5)	C5—C4—C3	118.5 (7)
S2—Cu2—S1^i^	97.69 (7)	C5—C4—H4	120.8
S2—Cu2—I2	114.82 (7)	C3—C4—H4	120.8
S1^i^—Cu2—I2	115.43 (6)	C4—C5—N1	120.0 (8)
S2—Cu2—I1	107.63 (7)	C4—C5—H5	120.0
S1^i^—Cu2—I1	110.49 (6)	N1—C5—H5	120.0
I2—Cu2—I1	110.04 (4)	N2—C6—C7	117.0 (7)
S2—Cu2—Cu1	120.62 (6)	N2—C6—S2	119.4 (5)
S1^i^—Cu2—Cu1	55.70 (5)	C7—C6—S2	123.3 (6)
I2—Cu2—Cu1	124.49 (4)	C8—C7—C6	120.0 (8)
I1—Cu2—Cu1	55.59 (3)	C8—C7—H7	120.0
S3—Cu3—S2^i^	110.21 (7)	C6—C7—H7	120.0
S3—Cu3—I3	114.83 (7)	C7—C8—C9	119.3 (8)
S2^i^—Cu3—I3	118.15 (7)	C7—C8—H8	120.4
S3—Cu3—S2^ii^	104.12 (8)	C9—C8—H8	120.4
S2^i^—Cu3—S2^ii^	92.93 (8)	C10—C9—C8	119.0 (8)
I3—Cu3—S2^ii^	113.75 (6)	C10—C9—H9	120.5
Cu1—I1—Cu2	65.52 (3)	C8—C9—H9	120.5
C1—N1—C5	124.8 (7)	C9—C10—N2	121.9 (8)
C1—N1—H1	117.6	C9—C10—H10	119.1
C5—N1—H1	117.6	N2—C10—H10	119.1
C10—N2—C6	122.8 (7)	N3—C11—C12	116.0 (7)
C10—N2—H2	118.6	N3—C11—S3	120.9 (6)
C6—N2—H2	118.6	C12—C11—S3	123.1 (6)
C11—N3—C15	123.5 (7)	C13—C12—C11	121.5 (8)
C11—N3—H3	118.3	C13—C12—H12	119.2
C15—N3—H3	118.3	C11—C12—H12	119.2
C1—S1—Cu1	117.6 (3)	C12—C13—C14	119.5 (8)
C1—S1—Cu2^i^	111.4 (2)	C12—C13—H13	120.3
Cu1—S1—Cu2^i^	125.91 (8)	C14—C13—H13	120.3
C1—S1—Cu1^i^	104.9 (3)	C15—C14—C13	119.7 (8)
Cu1—S1—Cu1^i^	74.47 (6)	C15—C14—H14	120.1
Cu2^i^—S1—Cu1^i^	72.26 (6)	C13—C14—H14	120.1
C6—S2—Cu3^i^	112.1 (3)	C14—C15—N3	119.7 (8)
C6—S2—Cu2	112.9 (3)	C14—C15—H15	120.1
Cu3^i^—S2—Cu2	126.77 (9)	N3—C15—H15	120.1
C6—S2—Cu3^iii^	98.9 (3)		
			
S3—Cu1—Cu2—S2	−164.32 (10)	S2^i^—Cu3—S3—Cu1	−1.65 (19)
S1—Cu1—Cu2—S2	−6.01 (13)	I3—Cu3—S3—Cu1	−138.06 (12)
S1^i^—Cu1—Cu2—S2	−77.56 (9)	S2^ii^—Cu3—S3—Cu1	96.91 (15)
I1—Cu1—Cu2—S2	91.21 (8)	S1—Cu1—S3—C11	162.0 (3)
Cu1^i^—Cu1—Cu2—S2	−45.38 (9)	S1^i^—Cu1—S3—C11	−89.3 (3)
S3—Cu1—Cu2—S1^i^	−86.77 (10)	I1—Cu1—S3—C11	37.7 (3)
S1—Cu1—Cu2—S1^i^	71.55 (11)	Cu2—Cu1—S3—C11	−32.9 (3)
I1—Cu1—Cu2—S1^i^	168.77 (6)	Cu1^i^—Cu1—S3—C11	−143.7 (3)
Cu1^i^—Cu1—Cu2—S1^i^	32.17 (6)	S1—Cu1—S3—Cu3	−17.16 (16)
S3—Cu1—Cu2—I2	12.56 (11)	S1^i^—Cu1—S3—Cu3	91.46 (15)
S1—Cu1—Cu2—I2	170.87 (8)	I1—Cu1—S3—Cu3	−141.50 (12)
S1^i^—Cu1—Cu2—I2	99.32 (7)	Cu2—Cu1—S3—Cu3	147.85 (12)
I1—Cu1—Cu2—I2	−91.91 (5)	Cu1^i^—Cu1—S3—Cu3	37.13 (17)
Cu1^i^—Cu1—Cu2—I2	131.49 (6)	C5—N1—C1—C2	0.8 (12)
S3—Cu1—Cu2—I1	104.47 (9)	C5—N1—C1—S1	179.9 (7)
S1—Cu1—Cu2—I1	−97.22 (9)	Cu1—S1—C1—N1	162.9 (5)
S1^i^—Cu1—Cu2—I1	−168.77 (6)	Cu2^i^—S1—C1—N1	−40.6 (7)
Cu1^i^—Cu1—Cu2—I1	−136.59 (5)	Cu1^i^—S1—C1—N1	−117.0 (6)
S3—Cu1—I1—Cu2	−117.13 (8)	Cu1—S1—C1—C2	−18.1 (8)
S1—Cu1—I1—Cu2	129.49 (7)	Cu2^i^—S1—C1—C2	138.4 (6)
S1^i^—Cu1—I1—Cu2	9.41 (5)	Cu1^i^—S1—C1—C2	61.9 (7)
Cu1^i^—Cu1—I1—Cu2	64.56 (7)	N1—C1—C2—C3	0.0 (12)
S2—Cu2—I1—Cu1	−115.48 (6)	S1—C1—C2—C3	−179.0 (7)
S1^i^—Cu2—I1—Cu1	−9.89 (5)	C1—C2—C3—C4	−0.9 (14)
I2—Cu2—I1—Cu1	118.74 (4)	C2—C3—C4—C5	1.1 (15)
S3—Cu1—S1—C1	−149.4 (3)	C3—C4—C5—N1	−0.3 (15)
S1^i^—Cu1—S1—C1	99.0 (3)	C1—N1—C5—C4	−0.7 (15)
I1—Cu1—S1—C1	−23.6 (3)	C10—N2—C6—C7	−4.2 (12)
Cu2—Cu1—S1—C1	48.0 (3)	C10—N2—C6—S2	169.9 (7)
Cu1^i^—Cu1—S1—C1	99.0 (3)	Cu3^i^—S2—C6—N2	15.5 (7)
S3—Cu1—S1—Cu2^i^	57.90 (12)	Cu2—S2—C6—N2	166.3 (5)
S1^i^—Cu1—S1—Cu2^i^	−53.73 (9)	Cu3^iii^—S2—C6—N2	−75.0 (6)
I1—Cu1—S1—Cu2^i^	−176.24 (7)	Cu3^i^—S2—C6—C7	−170.8 (6)
Cu2—Cu1—S1—Cu2^i^	−104.64 (10)	Cu2—S2—C6—C7	−20.0 (8)
Cu1^i^—Cu1—S1—Cu2^i^	−53.73 (9)	Cu3^iii^—S2—C6—C7	98.7 (7)
S3—Cu1—S1—Cu1^i^	111.63 (8)	N2—C6—C7—C8	2.8 (12)
S1^i^—Cu1—S1—Cu1^i^	0.0	S2—C6—C7—C8	−171.0 (7)
I1—Cu1—S1—Cu1^i^	−122.51 (6)	C6—C7—C8—C9	0.7 (14)
Cu2—Cu1—S1—Cu1^i^	−50.91 (9)	C7—C8—C9—C10	−3.2 (15)
S1^i^—Cu2—S2—C6	179.4 (3)	C8—C9—C10—N2	2.0 (15)
I2—Cu2—S2—C6	56.7 (3)	C6—N2—C10—C9	1.8 (14)
I1—Cu2—S2—C6	−66.2 (3)	C15—N3—C11—C12	−3.8 (11)
Cu1—Cu2—S2—C6	−126.1 (3)	C15—N3—C11—S3	175.5 (6)
S1^i^—Cu2—S2—Cu3^i^	−34.96 (14)	Cu3—S3—C11—N3	19.6 (7)
I2—Cu2—S2—Cu3^i^	−157.63 (10)	Cu1—S3—C11—N3	−159.8 (5)
I1—Cu2—S2—Cu3^i^	79.47 (13)	Cu3—S3—C11—C12	−161.2 (6)
Cu1—Cu2—S2—Cu3^i^	19.54 (16)	Cu1—S3—C11—C12	19.4 (8)
S1^i^—Cu2—S2—Cu3^iii^	68.67 (9)	N3—C11—C12—C13	1.5 (12)
I2—Cu2—S2—Cu3^iii^	−54.00 (9)	S3—C11—C12—C13	−177.8 (7)
I1—Cu2—S2—Cu3^iii^	−176.90 (5)	C11—C12—C13—C14	1.7 (14)
Cu1—Cu2—S2—Cu3^iii^	123.16 (7)	C12—C13—C14—C15	−2.8 (14)
S2^i^—Cu3—S3—C11	179.1 (3)	C13—C14—C15—N3	0.7 (14)
I3—Cu3—S3—C11	42.7 (3)	C11—N3—C15—C14	2.7 (13)
S2^ii^—Cu3—S3—C11	−82.3 (3)		

Symmetry codes: (i) −*x*+1, −*y*+1, −*z*+1; (ii) *x*−1, *y*, *z*; (iii) *x*+1, *y*, *z*.
